# Follow-up effects of exercise-based interventions on physical performance in cancer survivors: a systematic review and meta-analysis

**DOI:** 10.1007/s11764-026-02030-4

**Published:** 2026-05-02

**Authors:** Alexander M. Brooks, Brent Cunningham, Madelin R. Siedler, Sarah Burkart, Mark A. Sarzynski, Nathan H. Parker, Ciaran M. Fairman

**Affiliations:** 1 Department of Exercise Science, Arnold School of Public Health, University of South Carolina, Columbia, SC USA; 2 School of Human Movement and Nutrition Sciences, The University of Queensland, St Lucia, Brisbane, QLD Australia; 3 School of Health, University of Sunshine Coast, Sippy Downs, QLD Australia; 4 Department of Exercise and Health Science, College of Saint Benedict and Saint John’s University, Saint Joseph, MN USA; 5 Department of Health Outcomes and Behavior, H. Lee Moffitt Cancer Center and Research Institute, Tampa, FL USA

**Keywords:** Exercise oncology, Cancer survivorship, Cardiorespiratory fitness, Muscular strength, Walking capacity

## Abstract

**Purpose:**

Physical performance impairments are common in cancer survivors and can limit daily activities, quality of life, and long-term health. Although structured exercise programs have proven beneficial for improving physical performance, maintenance of these benefits is unclear. This study aimed to systematically evaluate whether improvements in physical performance are maintained following structured exercise oncology interventions.

**Methods:**

A systematic search was conducted for randomized controlled trials (RCTs) published between January 1990 and March 2025. Eligible trials engaged adult cancer survivors in structured exercise interventions and reported objective measures of cardiorespiratory fitness, muscular strength, and/or walking capacity at the end of the intervention and ≥ 3 months after program completion. Data were pooled using random-effects meta-analyses with weighted mean differences (WMD) used to summarize effects.

**Results:**

Twenty-four RCTs (2289 participants; mean follow-up post-intervention = 8 months) were included. Exercise significantly improved cardiorespiratory fitness at post-intervention (WMD =  + 1.76 ml/kg/min; *p* = 0.008); however, improvements were attenuated at follow-up (WMD =  + 1.24 ml/kg/min; *p* = 0.130). Similarly, upper and lower body strength improved post-intervention (WMD = + 3.35 kg; *p* = 0.001; WMD =  + 12.7 kg; *p* = 0.045), but effects diminished at follow-up (WMD =  + 1.80 kg; *p* = 0.081; WMD =  + 10.0 kg; *p* = 0.093). In contrast, walking capacity increased post-intervention (WMD = + 40.3 m; *p* = 0.002) and remained elevated at follow-up (WMD =  + 49.4 m; *p* = 0.006). Certainty of evidence ranged from very low to low across outcomes, primarily due to risk of bias, inconsistency, and imprecision in effect estimates.

**Conclusions:**

Structured exercise interventions were found to produce short-term improvements in physical performance among cancer survivors. Although gains in cardiorespiratory fitness and muscular strength appeared to persist at follow-up, they were attenuated compared with post-intervention and supported by very low certainty evidence. In contrast, walking capacity demonstrated sustained improvements at follow-up, though the certainty of evidence remained low. Future work is needed to identify longer-term effects (> 12 months) and develop strategies to better maintain improved physical performance.

**Implications for Cancer Survivors:**

While exercise programs can improve physical performance, these benefits may not persist without ongoing support. Cancer survivors should be encouraged to continue self-directed exercise after program completion, and exercise programs should incorporate strategies to maintain longer-term improvements in physical performance.

**Supplementary Information:**

The online version contains supplementary material available at https://doi.org/10.1007/s11764-026-02030-4.

## Introduction

The global population of individuals treated for cancer has grown steadily over the past several decades, with an estimated 20 million new cases diagnosed each year [1]. While advances in early detection and treatment have significantly improved survival rates, many individuals now face long-term health challenges that persist after treatment ends [2–6]. Among these challenges, impairments in physical performance, defined as the capacity to carry out physical tasks involving endurance, strength, and mobility, are especially prevalent and debilitating [7, 8]. Current evidence suggests that nearly half of long-term survivors (5 + years) report difficulty with activities essential for daily living, work, and recreation [7–10]. These self-reported limitations are also reflected in objective measures of physical performance, including reduced cardiorespiratory fitness [11], muscular strength [12], and walking capacity [13]. These impairments have critical health implications, as diminished physical performance is an independent predictor of poorer quality of life [14, 15], increased healthcare utilization [16], and higher cancer-specific and all-cause mortality [17–19].

Evidence from randomized controlled trials and subsequent meta-analyses has suggested that exercise interventions delivered before, during, and/or after treatment for cancer yield meaningful improvements in physical performance [20], quality of life [21], and other patient-reported outcomes [22]. However, these interventions are inherently time-limited, delivered predominantly under supervised conditions, and provide limited post-intervention strategies to support the maintenance of physical activity [23]. Due to this, evidence supporting the long-term maintenance of the benefits achieved during these interventions once supervision has been removed remains limited [24, 25]. Addressing this gap is essential, as the durability of exercise benefits has direct implications for survivorship care planning, the prevention of late effects, and the development of scalable, sustainable exercise models in oncology. Furthermore, without evidence of sustained benefits, it becomes difficult to justify the resources allocated to these programs or to design interventions that promote lasting improvements in physical performance and overall health outcomes among cancer survivors.

Systematic efforts to evaluate the long-term effects of exercise oncology programs on physical performance have been limited [26, 27]. Existing reviews have been confined to single follow-up time points (e.g., 6 months), examined single cancer types, or primarily focused on outcomes other than physical performance [26, 27]. There has also been limited attention given to the effects immediately following the intervention relative to follow-up, indicating how the benefits change over time. To address this gap, the present systematic review and meta-analysis defined follow-up as occurring ≥ 3 months after intervention completion, allowing for the inclusion of multiple time-points, and included a separate analysis of the effects immediately post-intervention. The objective was to determine the longer-term effects of exercise interventions on physical performance in cancer survivors. We hypothesized that the beneficial effects of exercise would diminish at follow-up and with increasing time since intervention completion.

## Methods

The protocol for this systematic review and meta-analysis was registered on the Open Science Framework (OSF) on April 1, 2025 (osf.io/nf5rk). The Cochrane Handbook for Systematic Reviews of Interventions and the Preferred Reporting Items for Systematic Reviews and Meta-Analyses (PRISMA) 2020 statement guidelines were used to guide the review’s conduct and reporting [28, 29].

### Search strategy

An electronic database search was conducted to identify peer-reviewed articles published between January 1990 and June 2024. The search was updated in March 2025 to identify any additional eligible trials published since the initial search. The databases searched included PubMed, EMBASE, Web of Science, and SPORTDiscus. The search strategy was tailored to each database; however, all included a combination of search terms relating to the key concepts: cancer, exercise, and follow-up. The Boolean operator “OR” was applied within each concept, and “AND” was used to connect the concepts. The references retrieved from the search were imported into Covidence (www.covidence.org), which is a web-based software platform for article screening. Titles and abstracts of identified trials were assessed for eligibility by two independent reviewers (AB and BC) with discrepancies resolved through discussion.

### Inclusion and exclusion criteria

Eligible trials included randomized controlled trials (RCTs) involving adults (≥ 18 years) diagnosed with cancer at any stage of the cancer care continuum (before, during, or after treatment). Trials were required to evaluate a structured exercise intervention, defined as planned, structured, and repetitive physical activity intended to maintain or improve physical fitness [30], and have a minimum duration of 6 weeks, as this timeframe has been shown to improve physical function in individuals with a history of cancer [31–33]. Interventions could be delivered in home, community, or clinical contexts either in-person or remotely but were required to include some form of supervision or adherence monitoring (e.g., in-person oversight, scheduled check-ins, or logbooks). Complementary components such as behavioral counseling, nutritional guidance, or dietary supplements were also permitted.

Comparator groups received no structured exercise intervention. Acceptable control conditions included standard medical care, minimal contact interventions (such as advice to increase physical activity or provision of educational materials), and general physical activity programs aimed at increasing daily movement (e.g., step count goals) without a prescribed exercise regimen. Non-exercise comparators involving light activities such as stretching or deep breathing were also acceptable, as these “placebo” conditions are increasingly used to control for study staff/participant interactions [34].

Trials were required to report at least one objective measure of physical performance, including cardiorespiratory fitness (measured or estimated VO₂peak), muscular strength (upper or lower limb one-repetition maximum, 1RM), or walking capacity (6-min walk test, 6MWT). In addition, at least one follow-up assessment conducted ≥ 3 months after completion of the exercise intervention was required. Although no universally accepted definition of follow-up exists in this field, the 3-month mark is frequently used in exercise oncology research and was selected as a pragmatic lower threshold. No maximum follow-up duration was specified.

Trials were excluded if they focused primarily on physical rehabilitation or joint mobilization, or if the intervention aimed solely to increase general physical activity (e.g., daily step counts) without delivering a structured exercise program. Crossover trials in which the control group received the intervention during the follow-up period were excluded, as this prevented valid between-group comparisons at follow-up. Trials were also excluded if participants received structured support during the follow-up period (e.g., scheduled counseling sessions or individualized exercise modifications), as these contacts reflect continued intervention. However, trials were eligible if participants were provided optional referrals or access to exercise facilities without active support from study staff.

### Data extraction

Data extraction was performed utilizing a standardized Excel spreadsheet by a single reviewer (AB) and was then reviewed for accuracy by a second team member (BC). Any discrepancies were resolved through discussion. The extracted data included study characteristics, participant demographics, and intervention details. Outcome data for eligible physical performance measures was recorded for baseline, post-intervention, and follow-up. In instances where multiple follow-up time points were reported (e.g., 6-month and 12-month follow-up), data was extracted from the longest reported follow-up time point to avoid dependencies. In manuscripts only reporting baseline and follow-up data, the original report describing post-intervention results was retrieved for inclusion. We preferentially extracted the mean, standard deviation (SD), and sample size for each outcome at baseline, post-intervention, and follow-up. When not available, we extracted the mean between group difference and 95% confidence interval for baseline- to post-intervention and baseline to follow-up. When these data were not reported, values were extracted from figures using the web application WebPlotDigitize (https://automeris.io/).

### Risk of bias

The Cochrane Risk of Bias (RoB) 2.0 tool was used to evaluate five specific domains of potential bias: (1) bias arising from the randomization process, (2) bias due to deviations from intended interventions, (3) bias due to missing outcome data, (4) bias in measurement of the outcome, and (5) bias in selection of the reported result [35]. Each domain was rated as “low risk,” “some concerns,” or “high risk,” and an overall risk of bias judgment was determined for each study. Risk of bias was assessed with respect to follow-up outcomes, as these represented the primary outcomes of interest in this review. Articles were reviewed independently by two reviewers (AB and BC), and any discrepancies were resolved through discussion.

### Data analysis

All statistical analyses were conducted using R (Version 2023.03.0). Analysis was based on the weighted mean difference (WMD) of change scores, calculated by subtracting the mean change from baseline to post-intervention or follow-up in the control group from the corresponding change in the intervention group. This approach was chosen over post-intervention or follow-up-only comparisons to account for potential baseline imbalances in trials with small sample sizes, in line with recommended best practices for meta-analysis of continuous outcomes [36–38].

For each study, the mean and standard deviation, or mean difference and 95% confidence interval, together with the sample size corresponding to the extracted data, were used to calculate between-group change scores and the SD of the change. As is common in published reports, most trials did not provide SDs of change scores. These were therefore imputed using the following formula:

$${SD}_{change}= \sqrt{{SD}_{baseline}^{2}+ {SD}_{\text{post or follow}-\mathrm{up}}^{2}-2 \times r \times {SD}_{\mathrm{baseline}} \times {SD}_{\text{post or follow}-\mathrm{up}}}$$

where *r* represents the correlation between baseline and post-intervention (or follow-up) measurements. We assumed *r* = 0.7, consistent with the moderate-to-strong correlations typically observed in repeated measures of physical performance outcomes (e.g., cardiorespiratory fitness, strength, and walk tests) [39–44]. We acknowledge that the true correlation may vary across trials and outcomes. To address this, sensitivity analyses were performed using values of *r* = 0.5 (lower correlation) and *r* = 0.9 (higher correlation) to assess whether the choice of correlation coefficient meaningfully influenced the results.

Random-effects meta-analysis with inverse-variance weighting was conducted in two stages. First, mean difference estimates for baseline-to-post-intervention changes relative to control were pooled to represent the immediate effects of the intervention. Second, mean difference estimates for baseline-to-follow-up changes relative to control were pooled at the longest available follow-up time point to assess the durability of effects over time. For trials with multiple eligible intervention arms (e.g., aerobic vs. resistance vs. control), groups were combined into a single comparison when sufficient summary information was provided as recommended by the Cochrane Handbook to avoid unit-of-analysis errors [45].

Statistical heterogeneity was assessed using the *I*^2^ statistic, with thresholds of 25%, 50%, and 75% interpreted as low, moderate, and high heterogeneity, respectively. To explore potential sources of heterogeneity, several a priori-defined subgroup and regression analyses were performed. Trials were grouped by treatment status, distinguishing interventions delivered during active treatment (e.g., surgery, chemotherapy, and radiotherapy) from those not delivered during active treatment. Follow-up analyses were additionally stratified by the time elapsed since intervention completion (≤ 6 months, 7–12 months, > 12 months).

In addition to subgroup analyses, meta-regression analyses were conducted to examine whether time since intervention completion as a continuous variable moderated the observed effects at follow-up. We also conducted a meta-regression to examine whether the magnitude of the post-intervention effect predicted the follow-up effect. This analysis was not included in the preregistration but was added to further clarify the potential relationship between immediate and sustained effects. All regression models were estimated using restricted maximum likelihood.

### Certainty of evidence

The certainty of evidence pertaining to each pooled estimate at follow-up was assessed using the GRADE (Grading of Recommendations Assessment, Development, and Evaluation) approach, as outlined in the Cochrane Handbook [46]. In brief, evidence from randomized controlled trials is rated as high certainty but may be downgraded for several reasons: risk of bias, inconsistency (heterogeneity), indirectness (differences between the study populations, interventions, comparators, or outcomes and those of interest), imprecision (wide confidence intervals or small sample sizes), and publication bias. Based on these factors, the certainty of evidence was classified as high**,** moderate, low, or very low. The full GRADE certainty of evidence profile for each outcome can be found in Supplementary File, Table 6.

## Results

**Fig. 1:**
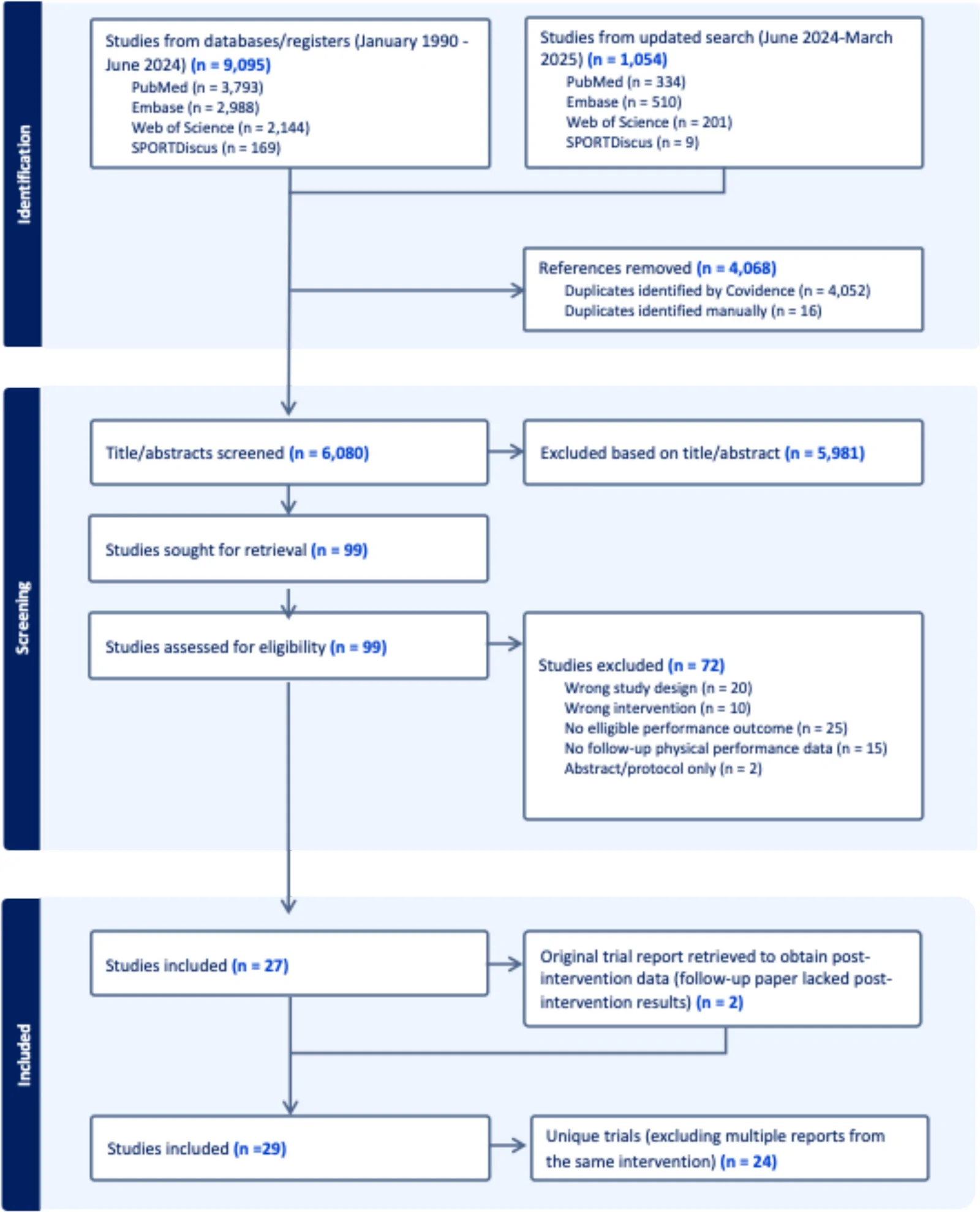
Flowchart illustrating the study selection process following the Preferred Reporting Items for Systematic Reviews and Meta-Analyses guidelines, including the number of records identified, screened, and included

The initial database search (up to June 2024) identified 9095 records, and an updated search (June 2024–March 2025) yielded an additional 1054 records. After removing 4068 duplicates, 6080 records were screened by title and abstract. Of these, 99 full-text articles were assessed for eligibility, with 72 excluded. A full list of excluded full texts, with reasons for exclusion, is provided in Supplementary File, Table 1. Ultimately, 27 publications met the inclusion criteria [47–73]. Two of these publications did not report post-intervention data and only provided baseline and follow-up results [56, 72]; therefore, the corresponding original reports were retrieved [74, 75]. The PACT trial enrolled individuals with both breast and colorectal cancer; however, results were published separately for these groups and were therefore treated as distinct trials in the analysis [70, 71]. In several cases, multiple publications originated from the same trial. In total, 29 publications were included in the review, representing 24 RCTs (Fig. 1).

### Participant and intervention characteristics

The included trials enrolled 2289 participants (*n* = 611 male, *n* = 1678 female) with mean ages ranging from 46 to 73 years. Most trials included individuals with stage I–III cancer, with two trials also including participants with stage IV disease [59, 64]. Participants represented a variety of cancer types, including breast [49–51, 54–56, 58, 67, 70], hematologic [61–63, 72], prostate [57, 64, 69], lung [52, 59], colorectal [53, 71], gynecological [60], esophagogastric [65], bladder [66], and mixed cancer cohorts [73]. Fourteen trials recruited participants not receiving active treatment [53, 55–58, 60, 61, 63, 65–67, 69, 72, 73], while ten included individuals undergoing treatment [49–52, 54, 59, 62, 64, 70, 71]. No trials were conducted exclusively in the pre-treatment (prehabilitation) setting.

Exercise interventions lasted 8 to 12 weeks [51–54, 56, 58, 59, 61–67] or 16 to 24 weeks [49, 50, 57, 60, 69–73], with one intervention lasting 1 year [55]. Programs included two to three exercise sessions per week, with the exception of one trial that prescribed one session per week [52]. Most programs combined aerobic exercise (AE) and resistance exercise (RE), while three trials used RE only [55, 67, 73], one used AE only [57], one combined AE with Pilates [53], and one combined RE or AE with high-intensity interval training (RE + HIIT or AE + HIIT) [49]. Interventions were primarily delivered in-person with supervision by physiotherapists or exercise specialists, with seven trials using virtual or mixed delivery formats [55–59, 63, 65]. Most trials included follow-ups at 3 to 6 months [50, 53, 57–59, 61, 64, 65, 67, 69–71, 73] or 7 to 12 months [51, 52, 54–56, 60, 62, 63, 66, 72], while only a single trial included follow-up beyond 12 months [49]. Intervention and follow-up characteristics are summarized in Table 1. A full summary of extracted study information can be found on the OSF repository (osf.io/nf5rk).

**Table 1:** Characteristics of included trials

Trial	Participants	Cancer type	Treatment status	Intervention length	Modality	Follow-up length (% lost to follow-up)
“Opti-Train Trial” Mijwel 2019 [47] Bolam 2019 [48] Anandavadivelan 2024 [49]	Ex: *n* = 159 Con: *n* = 81	Breast	Active treatment (adjuvant chemotherapy)	16 weeks	RE + HIIT Or AE + HIIT	56 months (61%)
Antunes 2023 [50]	Ex: *n* = 47 Con: *n* = 46	Breast	Active treatment (adjuvant chemotherapy)	20–24 weeks *(based on chemotherapy schedule)*	AE + RE	3 months (30%)
Bloomquist 2019 [51]	Ex: *n* = 75 Con: *n* = 78	Breast	Active treatment (adjuvant chemotherapy)	12 weeks	AE + RE	7 months (21%)
Brocki 2014 [52]	Ex: *n* = 41 Con: *n* = 37	Lung	Active treatment (mixed adjuvant therapy)	10 weeks	AE + RE	8 months (25%)
Cantarero-Villanueva 2016 [53]	Ex: *n* = 23 Con: *n* = 23	Colorectal	Post primary treatment	8 weeks	AE + Pilates	4 months (33%)
Chung 2022 [54]	Ex: *n* = 16 Con: *n* = 16	Breast	Active treatment (neo/adjuvant chemotherapy)	12 weeks	AE + RE	3 months (10%)
Dobek 2014 [55]	Ex: *n* = 52 Con: *n* = 54	Breast	Post primary treatment	52 weeks	RE	12 months (59%)
Dong 2019 [74] Dong 2020 [56]	Ex: *n* = 30 Con: *n* = 30	Breast	Post primary treatment	12 weeks	AE + RE	9 months (27%)
Eriksen 2017 [57]	Ex: *n* = 17 Con: *n* = 9	Prostate	Active surveillance	24 weeks	AE	6 months (27%)
Galiano-Castillo 2017 [58]	Ex: *n* = 40 Con: *n* = 41	Breast	Post primary treatment	8 weeks	AE + RE	4 months (12%)
Granger 2024 [59]	Ex: *n* = 58 Con: *n* = 58	Lung	Active treatment (mixed adjuvant therapy)	12 weeks	AE + RE	3 months (19%)
Hausmann 2018 [60]	Ex: *n* = 29 Con: *n* = 31	Gynecological	Post Primary Treatment	16 weeks	AE + RE	8 months (35%)
Knols 2010 [61]	Ex: *n* = 64 Con: *n* = 67	Leukemia and lymphoma	Post treatment (< 6 months post HSCT)	12 weeks	AE + RE	3 months (20%)
Larsen 2024 [62]	Ex: *n* = 54 Con: *n* = 46	Multiple myeloma	Active treatment (induction therapy)	10 weeks	AE + RE	10 months (23%)
Ma 2025 [63]	Ex: *n* = 73 Con: *n* = 66	Myeloma and lymphoma	Post treatment (≥ 6 months post HCT)	12 weeks	AE + RE	9 months (26%)
Ndjavera 2020 [64]	Ex: *n* = 24 Con: *n* = 26	Prostate	Active treatment (Initiating ADT)	12 weeks	AE + RE	3 months (26%)
O’Neil 2018 [65]	Ex: *n* = 21 Con: *n* = 22	Esophago-gastric	Post primary treatment	12 weeks	AE + RE	3 months (12%)
Porserud 2014 [66]	Ex: *n* = 9 Con: *n* = 9	Bladder	Post primary treatment	12 weeks	AE + RE	9 months (45%)
Rasmussen 2023 [67]	Ex: *n* = 10 Con: *n* = 10	Breast	Post primary treatment	12 weeks	RE	3 months (20%)
“RADAR Trial” Galvao 2014 [68] Taaffe 2018 [69]	Ex: *n* = 28 Con: *n* = 29 *Western Australia Site*	Prostate	Post primary treatment (ADT)	24 weeks	AE + RE	6 months (NR)
“PACT Trial” Travier 2015 [70]	Ex: *n* = 102 Con: *n* = 102	Breast	Active treatment (chemotherapy)	18 weeks	AE + RE	4.5 months (20%)
“PACT Trial” Van Vulpen 2016 [71]	Ex: *n* = 17 Con: *n* = 16	Colorectal	Active treatment (chemotherapy)	18 weeks	AE + RE	4.5 months (16%)
“EXIST Trial” Persoon 2017 [75] van Dongen 2019 [72]	Ex: *n* = 54 Con: *n* = 55	Hematologic	Post primary treatment	18 weeks	AE + RE	12 months (33%)
Winters-Stone 2023 [73]	Ex: *n* = 147 Con: *n* = 147	Mixed	Post primary treatment	24 weeks	RE	6 months (10%)

Follow-up is reported as months after intervention completion. Loss to follow-up is the percentage of participants enrolled in the study who did not complete the follow-up visit. Grouped trials: “Opti-Train” (Mijwel 2019, Bolam 2019, Anandavadivelan 2024), “RADAR” (Galvao 2014, Taaffe 2018), “EXIST” (Persoon 2017, van Dongen 2019), and “Dong et al.” (Dong 2019, Dong 2020) are presented together. The PACT trial (Travier 2015, Van Vulpen 2016) enrolled individuals with both breast and colorectal cancer; however, results were published separately for these groups with insufficient summary details to merge groups and were therefore treated as distinct trials in the analysis

*Ex* exercise, *Con* Control, *NR* not reported, *ADT* androgen deprivation therapy, *HSCT* hematopoietic stem cell transplant, *HCT* hematopoietic cell transplant, *RT-HIIT* resistance training plus high-intensity interval training, *AT-HIIT* aerobic training plus high-intensity interval training, *AE* aerobic exercise, *RE* resistance exercise

### Risk of bias

**Fig. 2:**
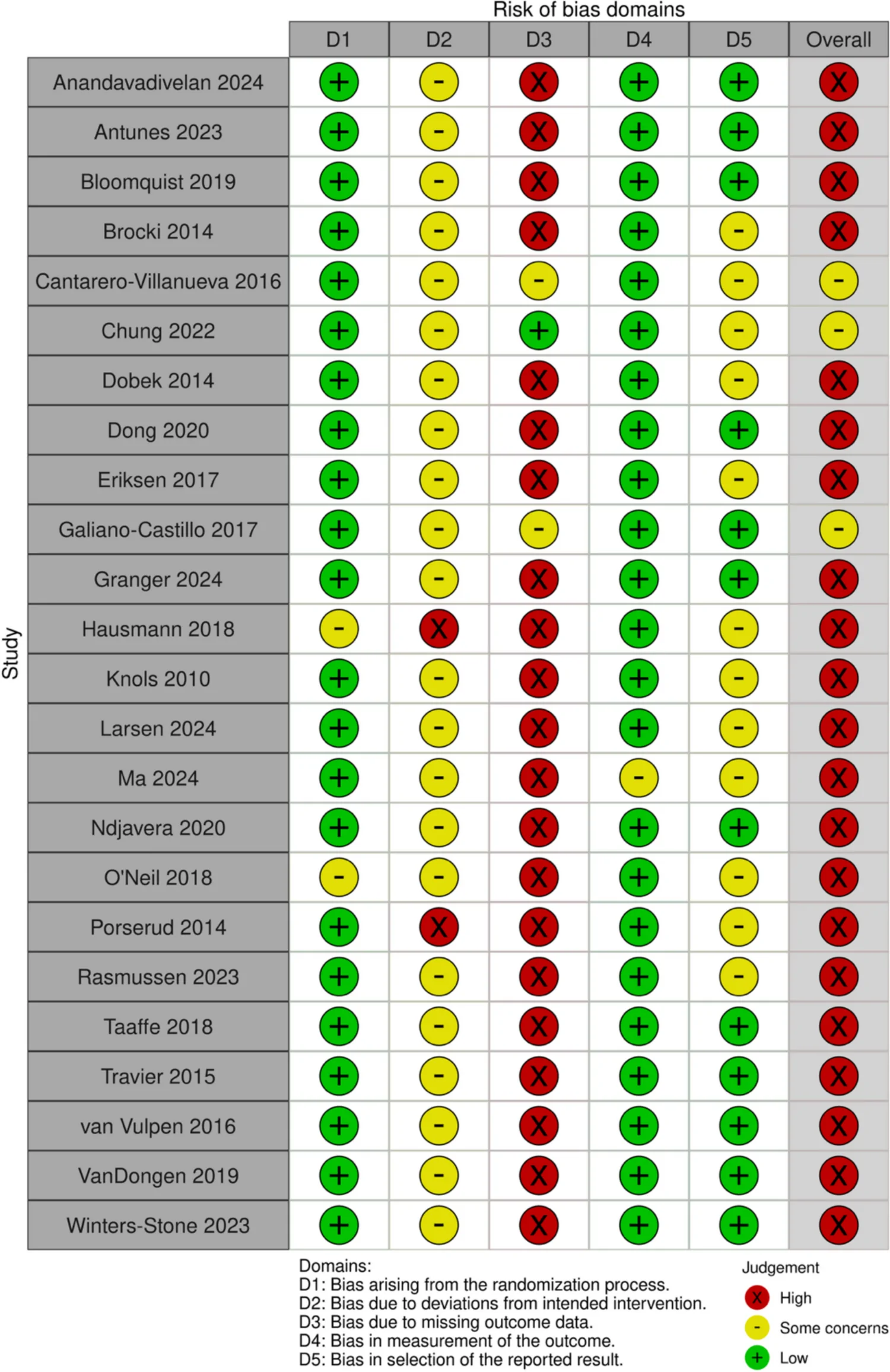
Cochrane Risk of Bias 2.0 assessments

Risk of bias assessments indicated that most trials were at high risk of bias overall for follow-up physical performance outcomes (Fig. 2). The randomization process (Domain 1) was generally well-conducted, with nearly all trials rated as low risk. However, deviations from intended interventions (Domain 2) were consistently rated as “some concerns,” reflecting the challenges of blinding participants and intervention staff in behavioral trials. The largest source of bias was missing outcome data (Domain 3), with the majority of trials rated as high risk due to attrition and incomplete follow-up without sufficient information to assess the potential impact on results. Measurement of outcomes (Domain 4) was largely low risk. For selective reporting (Domain 5), most trials were rated low risk or some concerns. Overall, 21 of 24 trials were judged to be at high risk of bias [49–52, 55–57, 59–67, 69–73], with the remainder judged as having some concerns [53, 54, 58].

### Cardiorespiratory fitness

**Fig. 3:**
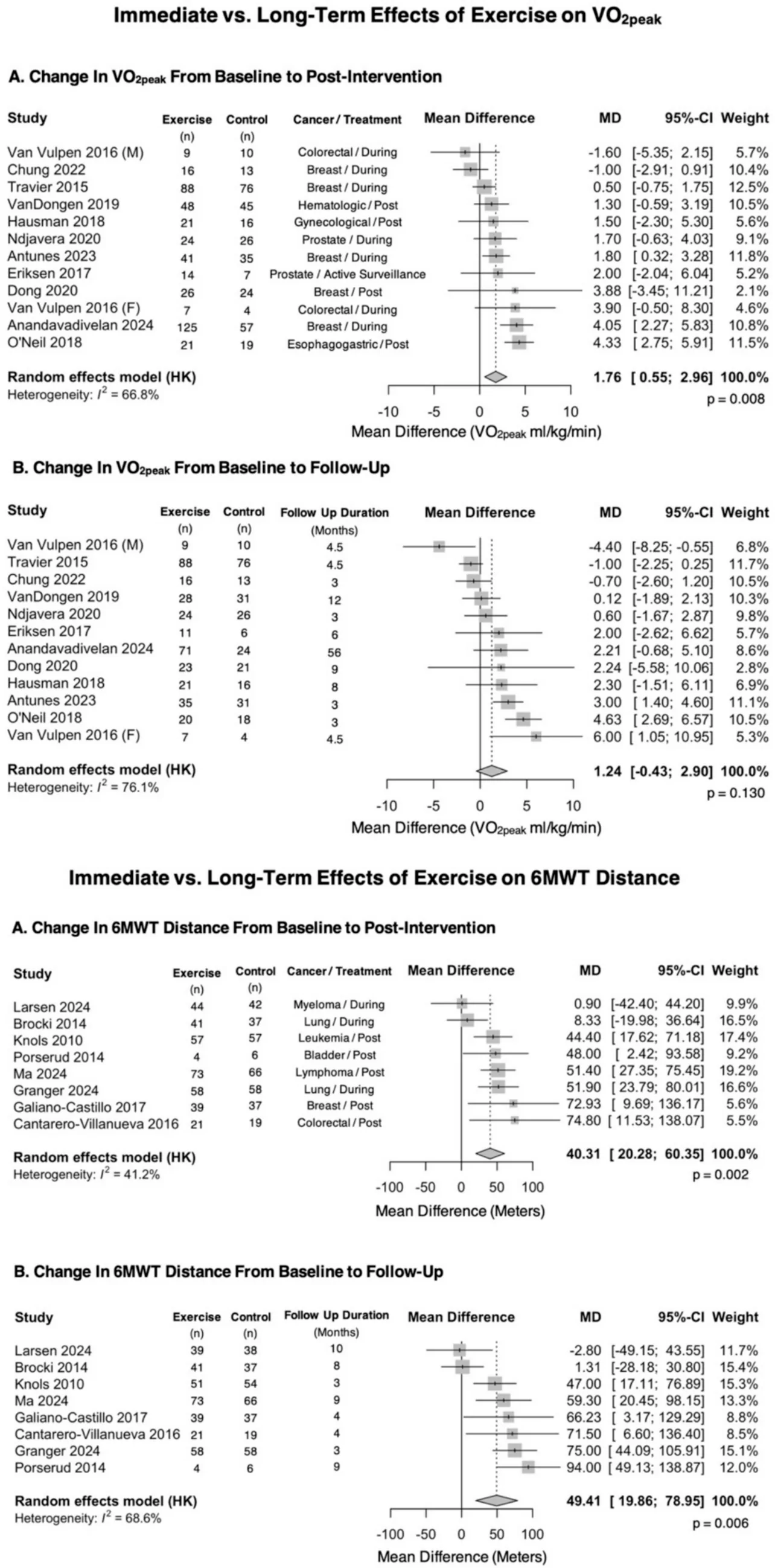
Forest plots displaying the between-group mean difference in VO2peak and 6MWT from **A** baseline to post-intervention and **B** baseline to follow-up

Eleven trials were included in the meta-analysis examining cardiorespiratory fitness (Fig. 3) [49, 50, 54, 56, 57, 60, 64, 65, 70–72]. From baseline to post-intervention (avg. = 3.7 months), exercise improved VO₂peak relative to control (WMD =  + 1.76 ml/kg/min; 95% CI, 0.55 to 2.96; *p* = 0.008), with moderate heterogeneity (*I*^2^ = 66.8%). At follow-up (avg. = 9.7 months post-intervention), improvements in VO₂peak were attenuated and not statistically significant (WMD =  + 1.24 ml/kg/min; 95% CI, –0.43 to 2.90; *p* = 0.130), with high heterogeneity (*I*^2^ = 76.1%). The certainty of evidence for follow-up effects was rated very low due to concerns about risk of bias, inconsistency, and imprecision.

### Walking capacity

Eight trials assessed walking capacity using the 6MWT (Fig. 3) [52, 53, 58, 59, 61–63, 66]. From baseline to post-intervention (avg. = 2.4 months), exercise improved walking capacity relative to control (WMD =  + 40.3 m; 95% CI 20.3–60.4; *p* = 0.002), with low-to-moderate heterogeneity (*I*^2^ = 41%). At follow-up (avg. = 6.3 months post-intervention), improvements were sustained (WMD =  + 49.4 m; 95% CI 19.9–79.0; *p* = 0.006), but heterogeneity was higher (*I*^2^ = 69%). The certainty of evidence for follow-up effects was rated low due to concerns about risk of bias and inconsistency.

### Upper-body strength

**Fig. 4:**
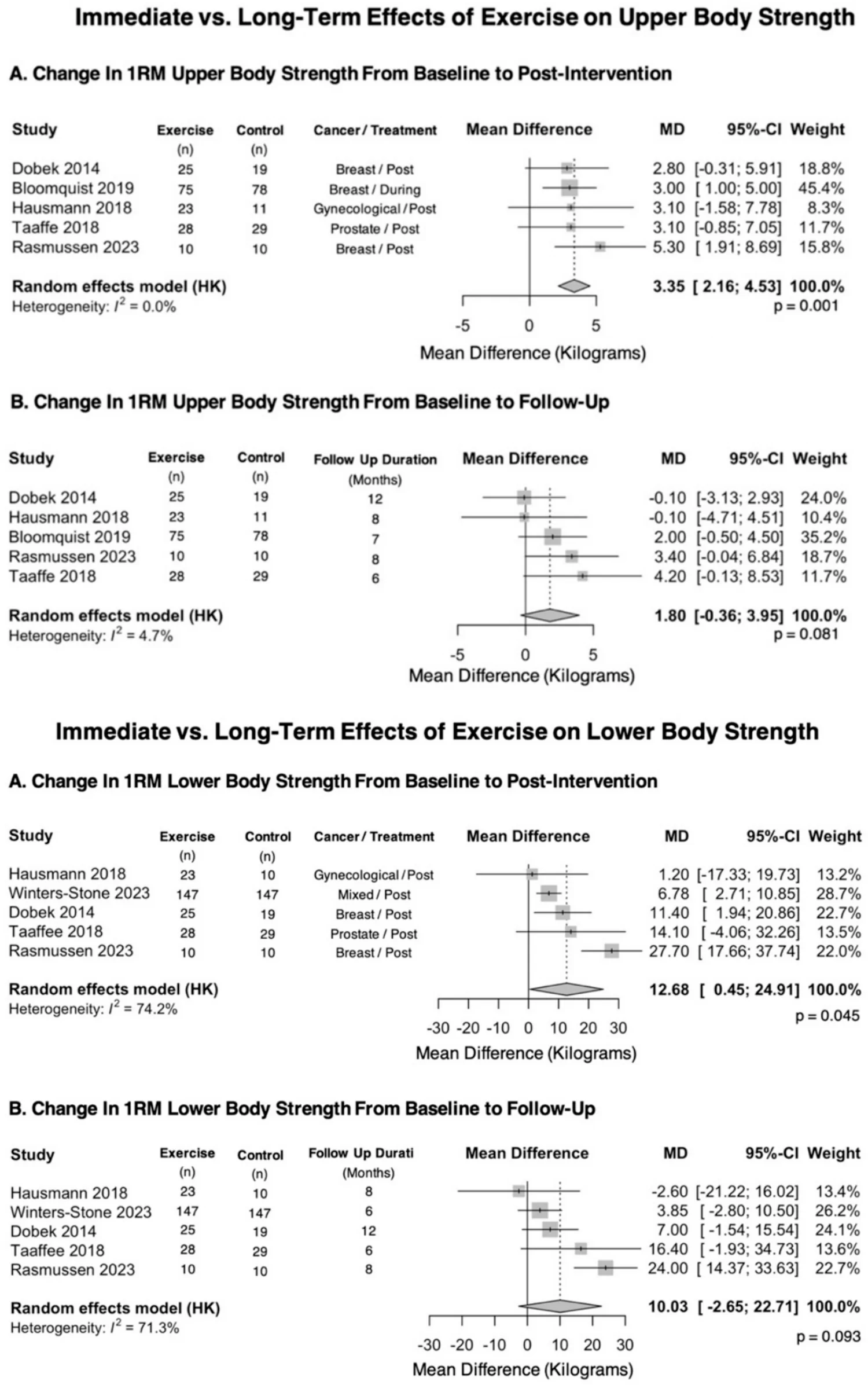
Forest plots displaying the between-group mean difference in upper-body and lower-body strength from **A** baseline to post-intervention and **B** baseline to follow-up

Five trials reported on upper-limb strength (Fig. 4) [51, 55, 60, 62, 67]. From baseline to post-intervention (avg. = 5.3 months), exercise improved upper-body strength relative to control (WMD =  + 3.35 kg; 95% CI 2.16 to 4.53; *p* = 0.0014), with no observed heterogeneity (*I*^2^ = 0%). At follow-up (avg. = 8.2 months post-intervention), improvements in upper-body strength were attenuated and not statistically significant (WMD =  + 1.80 kg; 95% CI –0.36 to 3.95; *p* = 0.08), with minimal heterogeneity (*I*^2^ = 4.7%). The certainty of evidence for follow-up effects was rated very low due to concerns about risk of bias and imprecision.

### Lower body strength

Five trials assessed lower-body strength (Fig. 4) [55, 60, 67, 69, 73]. From baseline to post-intervention (avg. = 5.9 months), exercise improved lower-body strength relative to control (WMD =  + 12.7 kg; 95% CI 0.45–24.9; *p* = 0.045), with high heterogeneity (*I*^2^ = 74%). At follow-up (avg. = 8.0 months post-intervention), improvements were attenuated and not statistically significant (WMD =  + 10.0 kg; 95% CI –2.65 to 22.7; *p* = 0.093), with similarly high heterogeneity (*I*^2^ = 71%). The certainty of evidence for follow-up effects was rated very low due to concerns about risk of bias, imprecision, and indirectness.

### Additional analysis

Subgroup analyses by treatment status and follow-up duration explained little of the observed heterogeneity across outcomes (Supplementary File, Tables 2–5). Although intervention effects generally appeared larger in participants not receiving active treatment and tended to decline with longer follow-up, none of these subgroup differences was statistically significant. An exception was the 6MWT, where post-treatment trials demonstrated minimal heterogeneity (*I*^2^ = 0%).

Meta-regression analyses did not identify a significant association between follow-up length and changes in outcomes (Supplementary File, Table 6). However, larger post-intervention improvements were strongly associated with larger follow-up improvements, explaining a substantial proportion of between-study variance (*R*^2^ ≈ 80–100%) (Supplementary File, Fig. 1).

Sensitivity analyses indicated that results were robust across r values (*r* = 0.5, 0.7, 0.9), with negligible differences in effect estimates and heterogeneity (Supplementary File, Tables 8–9).

## Discussion

Consistent with prior evidence, supervised exercise programs during and after cancer treatment produced improvements in cardiorespiratory fitness [76], walking capacity [77], and muscular strength [20]. At a follow-up of 3–12 months, most benefits persisted to some extent but were generally smaller in magnitude and no longer statistically significant, with the certainty of evidence rated very low to low. Walking capacity was an exception, with improvements maintained up to approximately 6 months post-intervention. Given the relatively short follow-up periods of the included trials (primarily < 12 months) and the low certainty of evidence for these estimates, it remains unclear whether these benefits would continue to decline or stabilize over longer durations.

To our knowledge, only two prior reviews have examined the longer-term effects of exercise interventions on physical performance in cancer survivors. Juvet et al. reported improvements in self-reported physical function among breast cancer survivors post-intervention but noted that standardized mean difference (SMD) began to diminish by 6 months follow-up (SMD, 0.27 to 0.19) [26]. In another review, Tan et al. were unable to meta-analyze strength outcomes due to heterogeneity in measurement methods. However, the authors reported sustained improvements in walking capacity (SMD = 0.62) and VO₂peak (WMD =  + 2.00 ml/kg/min) at 12 months of follow-up, although these estimates were based on only two and three trials, respectively, and the magnitude of the initial post-intervention improvements was not reported [27].

In the present review, the pooled analysis demonstrated a significant improvement in cardiorespiratory fitness post-intervention compared with control groups (+ 1.76 ml/kg/min). This effect is similar in magnitude to two prior meta-analyses of exercise interventions in cancer survivors by Sweegers et al. and Scott et al., which reported a WMD of + 1.80 and + 2.13 ml/kg/min, respectively [20, 76]. Importantly, this improvement exceeds the commonly used threshold of 1.35 ml/kg/min, which represents the technical error of VO₂peak assessments in cancer populations and has been used as the minimal detectable change above measurement error [39, 78]. Clinically, prior work in cancer survivors has suggested that each 3.5 ml/kg/min increase in VO₂peak is associated with significant reductions in all-cause (26%), cardiovascular (14%), and cancer-specific mortality (25%) [79, 80]. Furthermore, improvements as small as 1 ml/kg/min have been independently associated with reduced all-cause mortality, underscoring the potential prognostic relevance of even modest improvements [81].

At a mean follow-up of ~ 10 months, the overall effect on cardiorespiratory fitness relative to post-intervention diminished and was no longer statistically significant. Although meta-regression did not identify follow-up duration as a significant moderator, subgroup analyses suggested a pattern of diminishing effect over time, from + 1.16 ml/kg/min at 3–6 months to + 0.68 ml/kg/min at 7–12 months. However, these subgroup comparisons were not statistically significant and were based on a limited number of trials (eight and three, respectively), which restricts strong conclusions.

Similarly, exercise interventions improved muscular strength at post-intervention for both upper-body 1RM (+ 3.35 kg) and lower-body 1RM (+ 12.68 kg). These improvements are consistent with a prior meta-analysis of exercise interventions in cancer survivors by Sweegers et al. which reported a WMD in chest press strength of + 4.86 kg and leg press strength of + 16.56 kg [20]. Unlike cardiorespiratory fitness and walking capacity, there is not a widely accepted clinically important difference for strength measures in oncology. However, changes of this magnitude exceed typical test–retest variability (upper body ~ 1.8 kg, lower body ~ 5.5 kg) and may be functionally meaningful [82]. At a mean follow-up of ~ 8 months, strength gains were slightly attenuated relative to post-intervention and no longer statistically significant. Subgroup analyses and meta-regression did not identify follow-up duration as a significant moderator; however, these findings were limited by the small number of available trials (*n* = 5 for both upper- and lower-body strength).

Contrary to cardiorespiratory fitness and muscle strength, exercise interventions improved walking capacity as measured by 6MWT distance (+ 40.3 m), and this improvement was maintained at a mean follow-up of ~ 6 months (+ 49.4 m). This improvement exceeds the established minimal clinically important difference for cancer and chronic disease populations, which is generally estimated between 15 and 30 m [83], and has previously been associated with significant reductions (5–20%) in morbidity and mortality [84–87]. The sustained gains observed for walking capacity may reflect the relative accessibility of walking-based activities and the principles of specificity and reversibility in exercise adaptations, whereby without continued stimulus the adaptation will diminish [88]. Unlike structured resistance or aerobic exercise, walking can be easily incorporated into daily life without specialized equipment or facilities, which likely contributes to its durability. Walking has also previously been found to be the preferred mode of activity for many cancer survivors [89].

The pattern observed across outcomes is consistent with broader exercise physiology literature demonstrating that training adaptations decline following cessation of structured exercise, a phenomenon commonly referred to as detraining [90, 91]. Evidence from non-cancer populations suggests that cardiorespiratory fitness and muscular strength decline gradually during the first several months after training stops and may return toward baseline over longer periods if training is not resumed [91]. Given that most trials in our review assessed cardiorespiratory fitness and muscle strength at ≤ 12 months, it remains uncertain whether cancer survivors would follow a similar trajectory of eventual decline or whether partial gains might be sustained longer. This underscores the need for more long-term follow-up trials to determine whether improvements can be preserved in the absence of ongoing support or structured exercise.

Beyond physiological sustainability, maintenance of physical activity behavior appears challenging and may help explain the diminishing effects observed in the present study for cardiorespiratory fitness and muscle strength [23]. Of the included trials, Anandavadivelan et al. reported that the total number of minutes of moderate-to-vigorous physical activity each week measured via accelerometers decreased post-intervention and returned to baseline by follow-up, with no difference compared to the control group [49]. Similarly, Dobek et al. reported that at the 12-month follow-up visit, only 56% of participants in the exercise group reported that they had continued to exercise [55]. More broadly, evidence from other survivorship cohorts demonstrates similar patterns, with many participants failing to maintain recommended levels of physical activity once supervision ends [92–94].

These findings have important implications for survivorship care and the design of exercise interventions. Qualitative research suggests that many cancer survivors feel unprepared to maintain exercise once supervised programs end, often describing the transition as being “kicked out into the real world” without sufficient guidance or support [95–97]. Consistent with this, intervention studies indicate that incorporating targeted physical activity behavior change strategies may help support longer-term exercise participation in some [98–100], though not all [101, 102], contexts, highlighting the need to further refine best practices for sustaining engagement. Additionally, future interventions may benefit from explicitly planning for the transition from supervised to independent exercise. Approaches such as structured transition phases, reduced frequency “maintenance dose” programs, or periodic booster sessions may help preserve training adaptations while supporting survivors as they develop self-managed exercise routines.

An additional finding of the present review was that follow-up duration was not a significant moderator of outcomes. Although this result may partly reflect the limited number of available trials, it suggests that time since intervention completion alone may not be the primary determinant of sustained benefits. Instead, the magnitude of the initial post-intervention improvement emerged as a strong predictor of follow-up outcomes. This finding suggests that program characteristics that maximize early adaptations, such as training intensity, modality, or adherence, may ultimately play an important role in determining the extent to which benefits persist. Due to this, future research should consider moving beyond asking whether exercise benefits persist to asking under what conditions and with what program features they are most likely to be sustained.

Several limitations should be acknowledged when interpreting these findings. First, the majority of included trials assessed follow-up outcomes within 3–12 months of program completion. As a result, our findings primarily reflect short- to mid-term durability of exercise effects, and evidence on longer-term sustainability (beyond 1 year) remains sparse. Second, the majority of included trials were judged to be at high risk of bias, particularly in domains related to missing outcome data. The total number of participants from baseline to follow-up decreased by approximately 30%, with some trials reporting attrition rates of more than 50%. Such attrition is problematic because participants who discontinue or are lost to follow-up may be systematically different from those who remain. A final limitation is that most analyses were based on fewer than 10 trials. To obtain sufficient data for pooled effect estimates, trials involving different cancer types, treatment statuses, time since treatment completion, exercise interventions, and outcome assessments (e.g., estimated vs. measured VO2peak) were combined. While necessary for the meta-analysis, this approach likely contributed to the high levels of heterogeneity observed and further limits the certainty of the evidence, highlighting the need for additional trials with extended follow-up.

## Conclusion

This systematic review and meta-analysis demonstrated that supervised exercise interventions in cancer survivors produce meaningful short-term improvements in cardiorespiratory fitness, muscular strength, and walking capacity. However, these benefits diminished to some degree after program completion, with walking capacity showing the most sustained improvements. These findings highlight the importance of strategies to support long-term exercise participation to potentially maintain physiological improvements. Despite the short-term effects, limitations including high risk of bias, substantial loss to follow-up, and limited data beyond 1 year highlight the need for future research to evaluate the long-term benefits of exercise interventions on physical performance in cancer survivors.

## Supplementary Information

Below is the link to the electronic supplementary material.

ESM 1 (DOCX 752 KB)

## Data Availability

The datasets generated and analyzed during the current study are available in the open science repository, (osf.io/nf5rk).
