# Supplementary material for: Follow-up effects of exercise-based interventions on physical performance in cancer survivors: a systematic review and meta-analysis
Source: J Cancer Surviv. 2026 May 2;20(Suppl 2):2504–19. doi: 10.1007/s11764-026-02030-4 (PMC13744534; doi:10.1007/s11764-026-02030-4)
Supplement: Supplementary file 1 [file 11764_2026_2030_MOESM1_ESM.docx]

**Follow-Up Effects of Exercise-Based Interventions on Physical Performance in Cancer Survivors: A Systematic Review and Meta-Analysis**

Alexander M. Brooks^1^, Brent Cunningham^2,3^, Madelin R. Siedler^4^, Sarah Burkart^1^, Mark A. Sarzynski^1^, Nathan H. Parker^5^, Ciaran M. Fairman^1^

^1^ Department of Exercise Science, Arnold School of Public Health, University of South Carolina, Columbia, SC, USA

^2^ School of Human Movement and Nutrition Sciences, The University of Queensland, St Lucia, Queensland, Australia

^3^ School of Health, University of Sunshine Coast, Sippy Downs, Queensland, Australia

^4^ Department of Exercise and Health Science, College of Saint Benedict and Saint John's University, Saint Joseph, Minnesota, USA

^5^Department of Health Outcomes and Behavior, H. Lee Moffitt Cancer Center and Research Institute, Tampa, Florida, USA

**Corresponding Author:**

Alexander M. Brooks, M.S., CSCS

Arnold School of Public Health

University of South Carolina

921 Assembly Street, PHRC 220

Columbia, SC 29208

Email: amb91@email.sc.edu

Phone: 803-576-8404

Journal of Cancer Survivorship

**Table of Contents**

Page 2: Data extraction and analysis supplementary details

Page 3: Near misses

Page 4-7: Search strategy for each database

Page 8-10: Table 1. List of full-text articles excluded from the review

Page 11: Table 2. Treatment effect modifiers for changes in VO₂peak

Page 12: Table 3. Treatment effect modifiers for changes in upper body strength

Page 13: Table 4. Treatment effect modifiers for changes in lower body strength

Page 14: Table 5. Treatment effect modifiers for changes in 6MWT distance

Page 15: Table 6. Meta-regression examining the association between follow-up duration and changes in physical performance.

Page 16: Figure 1. Meta-regression examining the association between post-program and follow-up changes in physical performance

Page 17: Figure 2. Funnel plot results for Vo2peak

Page 18: Table 7. GRADE certainty of evidence profile

Page 19: Table 8. Sensitivity analysis of post-intervention WMD estimates using different correlation coefficients

Page 19: Table 9. Sensitivity analysis of follow-up WMD estimates using different correlation coefficients

Page 20: References

**Data Extraction and Analysis Supplementary Details**

*Cardiorespiratory Fitness*

Twelve studies were included in the meta-analysis examining cardiorespiratory fitness. Assessment methods included submaximal VO₂peak predictions (n = 3) and maximal cardiopulmonary exercise test derived VO₂peak (n = 9). Anandavadivelan et al. included two exercise arms (RE-HIIT and AE-HIIT), which were combined for analysis to form a single exercise group [1]. Van Vulpen et al. reported sex-specific results but did not provide post- or follow-up means and standard deviations [2]. Because the available data were limited to change scores and between-group mean differences with 95% CIs, the groups could not be merged and were retained as reported.

*Upper-Body Strength*

Five studies reported on upper-limb strength (three using 1RM chest press, two using 1RM bench press). Relevant data were not reported in Rasmussen et al. and were therefore extracted from a figure using the web application WebPlotDigitizer (<https://automeris.io/>) [3].

*Lower-Body Strength*

Five studies assessed lower-body strength (four using 1RM leg press, one using 1RM back squat). Winters-Stone et al. only reported baseline values and mean differences from baseline to post-intervention and from post-intervention to follow-up [4]. These data were used to impute the mean difference from baseline to follow-up for the pooled analysis. Relevant data were not reported in Rasmussen et al. and were therefore extracted using the web application WebPlotDigitizer (<https://automeris.io/>) [3].

Details of these calculations can be found in the data extraction spreadsheet on the open science repository (osf.io/nf5rk).

**Near Misses**

As is common with systematic reviews, there were several studies that could be considered “near-misses.” For example, the BEAT trial is a large RCT which reported on the long-term effects of exercise in individuals with cancer [5]. However, cardiorespiratory fitness in this trial was assessed using estimated submaximal oxygen consumption rather than direct or estimated peak oxygen consumption, which was required for inclusion. Similarly, the BREX study is another large RCT that reported long-term follow-up data, however, it employed the 2-km walk test rather than the 6-minute walk test [6]. Although studies such as these could have been included using standardized mean differences (SMDs), we prioritized weighted mean differences (WMDs) to preserve the original measurement units, enhance clinical interpretability, and minimize indirectness and heterogeneity.

**PubMed Search**

(("Bone Marrow Transplantation"[MeSH Terms] OR "Cancer Survivors"[MeSH Terms] OR "Neoplasms"[MeSH Terms] OR "Radiotherapy"[MeSH Terms] OR ("adenocarcinoma*"[Title/Abstract] OR "bone marrow transplantation*"[Title/Abstract] OR "cancer*"[Title/Abstract] OR "carcinoma*"[Title/Abstract] OR "hodgkin*"[Title/Abstract] OR "leukemia*"[Title/Abstract] OR "leukaemia*"[Title/Abstract] OR "lymphoma*"[Title/Abstract] OR "malignan*"[Title/Abstract] OR "melanoma*"[Title/Abstract] OR "mesothelioma*"[Title/Abstract] OR "metasta*"[Title/Abstract] OR "myeloma*"[Title/Abstract] OR "neoplas*"[Title/Abstract] OR "nonhodgkin*"[Title/Abstract] OR "oncolog*"[Title/Abstract] OR "osteosarcoma*"[Title/Abstract] OR "radiation therap*"[Title/Abstract] OR "Radiotherapy"[Title/Abstract] OR "sarcoma*"[Title/Abstract] OR "tumor*"[Title/Abstract] OR "tumour*"[Title/Abstract])) AND ("Dancing"[MeSH Terms] OR "Dance Therapy"[MeSH Terms] OR "Exercise"[MeSH Terms] OR "Exercise Therapy"[MeSH Terms] OR "Physical Fitness"[MeSH Terms] OR "Sports"[MeSH Terms] OR "Yoga"[MeSH Terms] OR ("aerobic training"[Title/Abstract] OR "dance movement"[Title/Abstract] OR "Dance Therapy"[Title/Abstract] OR "Dancing"[Title/Abstract] OR "endurance training"[Title/Abstract] OR "exercis*"[Title/Abstract] OR "high intensity interval"[Title/Abstract] OR "interval training"[Title/Abstract] OR "jogging"[Title/Abstract] OR "kinesiotherap*"[Title/Abstract] OR "muscle strengthening"[Title/Abstract] OR "physical activit*"[Title/Abstract] OR "Physical Fitness"[Title/Abstract] OR "physical training"[Title/Abstract] OR "pilates"[Title/Abstract] OR "pool therap*"[Title/Abstract] OR "power lifting"[Title/Abstract] OR "powerlifting"[Title/Abstract] OR "resistance training"[Title/Abstract] OR "Running"[Title/Abstract] OR "sport*"[Title/Abstract] OR "strength training"[Title/Abstract] OR "strengthening program"[Title/Abstract] OR "Swim"[Title/Abstract] OR "Swimming"[Title/Abstract] OR "t ai chi"[Title/Abstract] OR "tai ji"[Title/Abstract] OR "tai chi"[Title/Abstract] OR "Taijiquan"[Title/Abstract] OR "Walking"[Title/Abstract] OR "weight lifting"[Title/Abstract] OR "Weightlifting"[Title/Abstract] OR "weight training"[Title/Abstract] OR "Yoga"[Title/Abstract])) AND ("Follow-Up Studies"[MeSH Terms] OR "Longitudinal Studies"[MeSH Terms] OR ("Follow-up"[Title/Abstract] OR "followup"[Title/Abstract] OR "followups"[Title/Abstract] OR "follow-ups"[Title/Abstract] OR "post intervention"[Title/Abstract] OR "postintervention"[Title/Abstract] OR "longitudinal"[Title/Abstract]) OR (("12 week*"[Title/Abstract] OR "24 week*"[Title/Abstract] OR "36 week*"[Title/Abstract] OR "1 year"[Title/Abstract] OR "3 month*"[Title/Abstract] OR "6 month*"[Title/Abstract] OR "9 month*"[Title/Abstract] OR "12 month*"[Title/Abstract] OR "one year"[Title/Abstract] OR "three month*"[Title/Abstract] OR "six month*"[Title/Abstract] OR "nine month*"[Title/Abstract] OR "twelve month*"[Title/Abstract] OR "24 month*"[Title/Abstract] OR "twenty four month*"[Title/Abstract] OR "two year*"[Title/Abstract] OR "2 year*"[Title/Abstract] OR "3 year*"[Title/Abstract] OR "three years"[Title/Abstract] OR "5 year*"[Title/Abstract] OR "five year*"[Title/Abstract]) AND ("follow"[Title/Abstract] OR "following"[Title/Abstract] OR "followed"[Title/Abstract] OR "after intervention"[Title/Abstract] OR "sustain*"[Title/Abstract]))) AND (("randomized controlled trial"[Publication Type] OR "controlled clinical trial"[Publication Type] OR ("randomized"[Title/Abstract] OR "randomised"[Title/Abstract]) OR "placebo"[Title/Abstract] OR "drug therapy"[MeSH Subheading] OR "randomly"[Title/Abstract] OR "trial"[Title/Abstract] OR "groups"[Title/Abstract]) NOT ("animals"[MeSH Terms] NOT "humans"[MeSH Terms]))) AND (1990/1/1:2024/5/16[pdat])

**Embase Search**

('bone marrow transplantation'/exp OR 'cancer survivor'/exp OR 'neoplasm'/exp OR 'radiotherapy'/exp OR adenocarcinoma*:ti,ab OR 'bone marrow transplantation*':ti,ab OR cancer*:ti,ab OR carcinoma*:ti,ab OR hodgkin*:ti,ab OR leukemia*:ti,ab OR leukaemia*:ti,ab OR lymphoma*:ti,ab OR malignan*:ti,ab OR melanoma*:ti,ab OR mesothelioma*:ti,ab OR metasta*:ti,ab OR myeloma*:ti,ab OR neoplas*:ti,ab OR nonhodgkin*:ti,ab OR oncolog*:ti,ab OR osteosarcoma*:ti,ab OR 'radiation therap*':ti,ab OR radiotherapy:ti,ab OR sarcoma*:ti,ab OR tumor*:ti,ab OR tumour*:ti,ab) AND ('dancing'/exp OR 'dance therapy'/exp OR 'exercise'/exp OR 'kinesiotherapy'/exp OR 'fitness'/exp OR 'sport'/exp OR 'physical activity'/de OR 'walking'/de OR 'aerobic training':ti,ab OR 'dance movement':ti,ab OR 'dance therapy':ti,ab OR dancing:ti,ab OR 'endurance training':ti,ab OR exercis*:ti,ab OR 'high intensity interval':ti,ab OR 'interval training':ti,ab OR jogging:ti,ab OR kinesiotherap*:ti,ab OR 'muscle strengthening':ti,ab OR 'physical activit*':ti,ab OR 'physical fitness':ti,ab OR 'physical training':ti,ab OR pilates:ti,ab OR 'pool therap*':ti,ab OR 'power lifting':ti,ab OR powerlifting:ti,ab OR 'resistance training':ti,ab OR running:ti,ab OR sport*:ti,ab OR 'strength training':ti,ab OR 'strengthening program':ti,ab OR swim:ti,ab OR swimming:ti,ab OR 't ai chi':ti,ab OR 'tai ji':ti,ab OR 'tai chi':ti,ab OR taijiquan:ti,ab OR walking:ti,ab OR 'weight lifting':ti,ab OR weightlifting:ti,ab OR 'weight training':ti,ab OR yoga:ti,ab) AND ('follow up'/exp OR 'longitudinal study'/de OR 'follow-up':ti,ab OR followup:ti,ab OR followups:ti,ab OR 'follow-ups':ti,ab OR 'post intervention':ti,ab OR postintervention:ti,ab OR longitudinal:ti,ab OR (('12 week*':ti,ab OR '24 week*':ti,ab OR '36 week*':ti,ab OR '1 year':ti,ab OR '3 month*':ti,ab OR '6 month*':ti,ab OR '9 month*':ti,ab OR '12 month*':ti,ab OR 'one year':ti,ab OR 'three month*':ti,ab OR 'six month*':ti,ab OR 'nine month*':ti,ab OR 'twelve month*':ti,ab OR '24 month*':ti,ab OR 'twenty four month*':ti,ab OR 'two year*':ti,ab OR '2 year*':ti,ab OR '3 year*':ti,ab OR 'three year*':ti,ab OR '5 year*':ti,ab OR 'five year*':ti,ab) AND (follow:ti,ab OR following:ti,ab OR followed:ti,ab OR 'after intervention':ti,ab OR sustain*:ti,ab))) AND ('crossover procedure':de OR 'double-blind procedure':de OR 'randomized controlled trial':de OR 'single-blind procedure':de OR random*:de,ab,ti OR factorial*:de,ab,ti OR crossover*:de,ab,ti OR ((cross NEXT/1 over*):de,ab,ti) OR placebo*:de,ab,ti OR ((doubl* NEAR/1 blind*):de,ab,ti) OR ((singl* NEAR/1 blind*):de,ab,ti) OR assign*:de,ab,ti OR allocat*:de,ab,ti OR volunteer*:de,ab,ti) AND [1990-2024]/py AND ('article'/it OR 'article in press'/it)

**SPORTDiscus Search**

((DE "Cancer" OR DE "Cancer Survivors" OR "CANCER in young adults" OR "LUNG cancer" OR "CANCER survivors") OR (TI OR AB OR SU (Adenocarcinoma* OR "bone marrow transplantation*" OR Cancer* OR Carcinoma* OR Hodgkin* OR Leukemia* OR Leukaemia* OR Lymphoma* OR Malignan* OR Melanoma* OR Mesothelioma* OR Metasta* OR Myeloma* OR Neoplas* OR Nonhodgkin* OR Oncolog* OR Osteosarcoma* OR "Radiation therap*" OR radiotherapy OR Sarcoma* OR Tumor* OR Tumour*))) AND ((DE "EXERCISE" OR DE "EXERCISE therapy" OR DE "PHYSICAL fitness" OR DE "SPORTS" OR DE "WALKING" OR DE "YOGA") OR (TI OR AB OR SU ("Aerobic training" OR "Dance movement" OR "Dance therapy" OR Dancing OR "Endurance training" OR Exercis* OR "High intensity interval" OR "interval training" OR Jogging OR kinesiotherap* OR "Muscle strengthening" OR "Physical activit*" OR "Physical fitness" OR "physical training" OR Pilates OR "Pool therap*" OR "Power lifting" OR powerlifting OR "Resistance training" OR Running OR Sport* OR "Strength training" OR "Strengthening program" OR Swim OR Swimming OR "T’ai chi" OR "Tai ji" OR "Tai chi" OR Taijiquan OR Walking OR "Weight lifting" OR Weightlifting OR "Weight training" OR Yoga))) AND ((TI OR AB OR SU ("Follow-up" OR followup OR followups OR "follow-ups" OR "post intervention" OR postintervention OR longitudinal)) OR (TI OR AB OR SU (("12 week*" OR "24 week*" OR "one year" OR "3 month*" OR "6 month*" OR "9 month*" OR "12 month*" OR "1 year" OR "three month*" OR "six month*" OR "nine month*" OR "twelve month*" OR "24 month*" OR "twenty four month*" OR "two year*" OR "2 year*" OR "3 year*" OR "three years" OR "5 year*" OR "five year*") AND (follow OR following OR followed OR "after intervention" OR sustain*)))) AND ((DE "Randomized Controlled Trials") OR (TI OR AB (randomised OR randomized OR randomisation OR randomization OR placebo* OR (random* AND (allocat* OR assign*)) OR (blind* AND (single OR double OR treble OR triple))))) Limiters: Peer-reviewed; Published January 1990–May 2024.

**Web of Science Search**

((TI=(Adenocarcinoma* OR "bone marrow transplantation*" OR Cancer* OR Carcinoma* OR Hodgkin* OR Leukemia* OR Leukaemia* OR Lymphoma* OR Malignan* OR Melanoma* OR Mesothelioma* OR Metasta* OR Myeloma* OR Neoplas* OR Nonhodgkin* OR Oncolog* OR Osteosarcoma* OR "Radiation therap*" OR radiotherapy OR Sarcoma* OR Tumor* OR Tumour*) OR AB=(Adenocarcinoma* OR "bone marrow transplantation*" OR Cancer* OR Carcinoma* OR Hodgkin* OR Leukemia* OR Leukaemia* OR Lymphoma* OR Malignan* OR Melanoma* OR Mesothelioma* OR Metasta* OR Myeloma* OR Neoplas* OR Nonhodgkin* OR Oncolog* OR Osteosarcoma* OR "Radiation therap*" OR radiotherapy OR Sarcoma* OR Tumor* OR Tumour*)) AND (TI=("Aerobic training" OR "Dance movement" OR "Dance therapy" OR Dancing OR "Endurance training" OR Exercis* OR "High intensity interval" OR "interval training" OR Jogging OR kinesiotherap* OR "Muscle strengthening" OR "Physical activit*" OR "Physical fitness" OR "physical training" OR Pilates OR "Pool therap*" OR "Power lifting" OR powerlifting OR "Resistance training" OR Running OR Sport* OR "Strength training" OR "Strengthening program" OR Swim OR Swimming OR "T’ai chi" OR "Tai ji" OR "Tai chi" OR Taijiquan OR Walking OR "Weight lifting" OR Weightlifting OR "Weight training" OR Yoga) OR AB=("Aerobic training" OR "Dance movement" OR "Dance therapy" OR Dancing OR "Endurance training" OR Exercis* OR "High intensity interval" OR "interval training" OR Jogging OR kinesiotherap* OR "Muscle strengthening" OR "Physical activit*" OR "Physical fitness" OR "physical training" OR Pilates OR "Pool therap*" OR "Power lifting" OR powerlifting OR "Resistance training" OR Running OR Sport* OR "Strength training" OR "Strengthening program" OR Swim OR Swimming OR "T’ai chi" OR "Tai ji" OR "Tai chi" OR Taijiquan OR Walking OR "Weight lifting" OR Weightlifting OR "Weight training" OR Yoga)) AND ((TI=("Follow-up" OR followup OR followups OR "follow-ups" OR "post intervention" OR postintervention OR longitudinal) OR AB=("Follow-up" OR followup OR followups OR "follow-ups" OR "post intervention" OR postintervention OR longitudinal) OR TI=(("12 week*" OR "24 week*" OR "one year" OR "3 month*" OR "6 month*" OR "9 month*" OR "12 month*" OR "1 year" OR "three month*" OR "six month*" OR "nine month*" OR "twelve month*" OR "24 month*" OR "twenty four month*" OR "two year*" OR "2 year*" OR "3 year*" OR "three years" OR "5 year*" OR "five year*") AND (follow OR following OR followed OR "after intervention" OR sustain*)) OR AB=(("12 week*" OR "24 week*" OR "one year" OR "3 month*" OR "6 month*" OR "9 month*" OR "12 month*" OR "1 year" OR "three month*" OR "six month*" OR "nine month*" OR "twelve month*" OR "24 month*" OR "twenty four month*" OR "two year*" OR "2 year*" OR "3 year*" OR "three years" OR "5 year*" OR "five year*") AND (follow OR following OR followed OR "after intervention" OR sustain*)))) AND (TS=(randomised OR randomized OR randomisation OR randomization OR placebo* OR (random* AND (allocat* OR assign*)) OR (blind* AND (single OR double OR treble OR triple))))) Limits applied: Peer-reviewed publications; Date range: January 1, 1990 – May 16, 2024.

| **Table 1.** List of full-text articles which were excluded from the review. | | |
| --- | --- | --- |
| **Author** | **Year** | **Exclusion Reason** |
| Mutrie | 2007 | Does not include eligible performance outcome. |
| Mutrie | 2012 | Does not include eligible performance outcome. |
| Bourke | 2011 | Does not include eligible performance outcome. |
| Cantarero-Villanueva | 2013 | Does not include eligible performance outcome. |
| Carayol | 2019 | Does not include eligible performance outcome. |
| Lin | 2022 | Does not include eligible performance outcome. |
| Lozano-Lozano | 2020 | Does not include eligible performance outcome. |
| Vehmanen | 2021 | Does not include eligible performance outcome. |
| Zhao | 2024 | Does not include eligible performance outcome. |
| Galiano-Castillo | 2016 | Does not include eligible performance outcome. |
| Rogers | 2015 | Does not include eligible performance outcome. Vo2 is estimation of submaximal oxygen consumption. |
| Rogers | 2016 | Does not include eligible performance outcome. Vo2 is estimation of submaximal oxygen consumption. |
| Rogers | 2023 | Does not include eligible performance outcome. Vo2 is estimation of submaximal oxygen consumption. |
| Rogers | 2009 | Does not include eligible performance outcome. Vo2 is estimation of submaximal oxygen consumption. |
| Winters-Stone | 2022 | Only reports eligible performance outcome at pre and post and not at follow-up. |
| Donnelly | 2011 | Non exercise-based intervention (advice to increase physical activity/steps). |
| Frensham | 2018 | Non exercise-based intervention (advice to increase physical activity/steps). |
| Alvarez-Salvago | 2023 | This study pooled long-term follow-up data from two previously published trials but only reported outcomes at baseline and ≥5-year follow-up. |
| Edbrooke | 2019 | Received monthly calls to review and progress exercise programs during the follow-up period. |
| Anderson | 2012 | Received monthly calls to review and progress exercise programs during the follow-up period. |
| Casla | 2015 | Crossover design in which the control group received the intervention during follow up. |
| Furzer | 2016 | Crossover design in which the control group received the intervention during follow up. |
| Milne | 2008 A | Crossover design in which the control group received the intervention during follow up. |
| Milne | 2008 B | Crossover design in which the control group received the intervention during follow up. |
| Newton | 2020 | Crossover design in which the control group received the intervention during follow up. |

| **Table 1.** Continued… | | |
| --- | --- | --- |
| **Author** | **Year** | **Exclusion Reason** |
| Schneider | 2023 | Crossover design in which the control group received the intervention during follow up. |
| Taaffe | 2017 | Crossover design in which the control group received the intervention during follow up. |
| Dieli-Conwright | 2018 A | Only the exercise group was followed up with after the intervention. |
| Dieli-Conwright | 2021 | Only the exercise group was followed up with after the intervention. |
| Dieli-Conwright | 2018 B | Only the exercise group was followed up with after the intervention. |
| Sheehan | 2020 | Only the exercise group was followed up with after the intervention. |
| Cantwell | 2024 | Both groups received supervised exercise training (intervention received additional online support). |
| Kilbreath | 2011 | Exercise intervention consisted of primarily shoulder rehabilitation and stretching following surgery. |
| Zhang | 2025 | Exercise intervention consisted of primarily shoulder rehabilitation and stretching following surgery. |
| Satish | 2024 | Follow up duration was < 3 months. |
| Zimmer | 2018 | Follow up duration was < 3 months (4 weeks). |
| Hayes | 2023 | Follow up duration was < 3 months (8 weeks). |
| Schmitz | 2010 | Intervention included 13 weeks supervised and 9 months unsupervised. However, assessments only occurred at baseline and 12 months. |
| Mustian | 2009 | Intervention is too short (4 weeks) and needs to be at least 6. |
| Thoft Jensen | 2023 | Intervention is too short (2 weeks) and needs to be at least 6. |
| Adams | 2018 | No follow up assessment of physical performance was performed (only surveys). |
| Courneya | 2009 | No follow up assessment of physical performance was performed (only surveys). |
| Schulz | 2022 | No follow up assessment of physical performance was performed (only surveys). |
| van Vulpen | 2021 | No follow up assessment of physical performance was performed (only surveys). |
| Haines | 2010 | No follow up assessment of physical performance was performed (only surveys). |
| Dillon | 2025 | No follow up assessments were performed. |
| Lee | 2019 | No follow up assessments were performed. |
| Schwarz | 2009 | No follow up assessments were performed. |
| Courneya | 2007 | No follow up assessments were performed. |
| Foulkes | 2023 | No follow up assessments were performed. |

| **Table 1.** Continued… | | |
| --- | --- | --- |
| **Author** | **Year** | **Exclusion Reason** |
| Kamel | 2020 | No follow up assessments were performed. |
| Mijwel | 2018 | No follow up assessments were performed. |
| Segal | 2009 | No follow up assessments were performed. |
| Gallardo-Rodriguez | 2023 | No follow up assessments were performed. |
| Baumann | 2017 | No physical performance outcomes. |
| Berglund | 1994 | No physical performance outcomes. |
| Courneya | 2012 | No physical performance outcomes. |
| Courneya | 2012 | No physical performance outcomes. |
| Guinan | 2013 | No physical performance outcomes. |
| Lin | 2023 | No physical performance outcomes. |
| Martin | 2016 | No physical performance outcomes. |
| Poort | 2020 | No physical performance outcomes. |
| Ansund | 2021 | No physical performance outcomes. |
| Penttinen | 2019 | No physical performance outcomes. |
| Blomqvist | 2020 | Non empirical publication (abstract). |
| Sharma | 2025 | Non empirical publication (abstract). |
| Buchan | 2016 | No non-exercise control group (aerobic vs. resistance exercise). |
| Simonsen | 2020 | Physical performance was only acessed in the exercise group and not in the control group. |
| Atkinson | 2021 | Study population included individuals < 18 years of age. |
| Fong | 2014 | Not a randomized control trial. Participants self-selected intervention group. |
| Hillengass | 2024 | Not a randomized control trial. Participants self-selected intervention group. |
| Houben | 2024 | Not a randomized control trial. Participnats in control group were all from a separate study site. |

| **Table 2.** Treatment effect modifiers for changes in VO_2_peak. | | | | | | | | | |
| --- | --- | --- | --- | --- | --- | --- | --- | --- | --- |
| Intervention Modifier | **WMD** | | | | | | | | |
|  | **^a^Pre-Post** | | | |  | **^b^Pre-Follow Up** | | | |
|  | No. of Studies | Mean (95% CI), direction | I^2^ | p |  | No. of Studies | Mean (95% CI), direction | I^2^ | p |
| **Treatment Status** |  |  |  |  |  |  |  |  |  |
| No Modifier (Reference) | 12 | +1.76 (0.55 to 2.96), Ex | 66.8% |  |  | 12 | +1.24 (-0.43 to 2.90), Ex | 76.1% |  |
| Active Treatment | 7 | +1.31 (-0.57 to 3.20), Ex | 70.7% |  |  | 7 | +0.65 (-2.07 to 3.37), Ex | 79.1% |  |
| Post Primary Treatment | 5 | +2.63 (0.71 to 4.54), Ex | 40.2% |  |  | 5 | +2.31 (-0.14 to 4.76), Ex | 60.1% |  |
| Test For Subgroup Differences |  |  |  | 0.2033 |  |  |  |  | 0.2410 |
| **Follow-Up Duration** |  |  |  |  |  |  |  |  |  |
| 3-6 Months | - | - | - |  |  | 8 | +1.16 (-1.48 to 3.80), Ex | 84.1% |  |
| 7-12 Months | - | - | - |  |  | 3 | +0.68 (-2.21 to 3.56), Ex | 0% |  |
| >12 Months | - | - | - |  |  | 1 | +2.2 (-0.68 to 5.10), Ex | n/a |  |
| Test For Subgroup Differences |  |  |  |  |  |  |  |  | 0.6307 |
| ^a^ Represents the MD in VO_2_peak (ml/kg/min) between the exercise and control group at the end of the exercise intervention.  ^b^ Represents the MD in VO_2_peak (ml/kg/min) between the exercise and control group at the end of the follow-up period.  Abbreviations: Ex, favors exercise | | | | | | | | | |

| **Table 3.** Treatment effect modifiers for changes in upper body strength. | | | | | | | | | |
| --- | --- | --- | --- | --- | --- | --- | --- | --- | --- |
| Intervention Modifier | **WMD** | | | | | | | | |
|  | **^a^Pre-Post** | | | |  | **^b^Pre-Follow Up** | | | |
|  | No. of Studies | Mean (95% CI), direction | I^2^ | p |  | No. of Studies | Mean (95% CI), direction | I^2^ | p |
| **Treatment Status** |  |  |  |  |  |  |  |  |  |
| No Modifier (Reference) | 5 | +3.35 (2.16 to 4.53), Ex | 0.0% |  |  | 5 | +1.80 (-0.36 to 3.95), Ex | 4.7% |  |
| Active Treatment | 1 | +3.00 (1.00 to 5.00), EX | n/a |  |  | 1 | + 2.00 (-0.50 to 4.50), Ex | n/a |  |
| Post Primary Treatment | 4 | +3.63 (1.67 to 5.60), EX | 0.0% |  |  | 4 | +1.76 (-1.80 to 5.31), Ex | 27.9% |  |
| Test For Subgroup Differences |  |  |  | 0.5948 |  |  |  |  | 0.8864 |
| **Follow-Up Duration** |  |  |  |  |  |  |  |  |  |
| 3-6 Months | - | - | - |  |  | 1 | +4.20 (-0.13 to 8.53), Ex | n/a |  |
| 7-12 Months | - | - | - |  |  | 4 | +1.48 (-1.03 to 3.98), Ex | 0.0% |  |
| >12 Months | - | - | - |  |  | - | - | - |  |
| Test For Subgroup Differences |  |  |  |  |  |  |  |  | 0.2463 |
| ^a^ Represents the MD in upper body strength (1repetition max, kg) between the exercise and control group at the end of the exercise intervention.  ^b^ Represents the MD in upper body strength (1repetition max, kg) between the exercise and control group at the end of the follow-up period.  Abbreviations: Ex, favors exercise | | | | | | | | | |

| **Table 4.** Treatment effect modifiers for changes in lower body strength. | | | | | | | | | |
| --- | --- | --- | --- | --- | --- | --- | --- | --- | --- |
| Intervention Modifier | **WMD** | | | | | | | | |
|  | **^a^Pre-Post** | | | |  | **^b^Pre-Follow Up** | | | |
|  | No. of Studies | Mean (95% CI), direction | I^2^ | p |  | No. of Studies | Mean (95% CI), direction | I^2^ | p |
| **Treatment Status** |  |  |  |  |  |  |  |  |  |
| No Modifier (Reference) | 5 | +12.68 (0.45 to 24.91), Ex | 74.2% |  |  | 5 | +10.03 (-2.65 to 22.71), Ex | 71.3% |  |
| Active Treatment | 0 | n/a | n/a |  |  | 0 | n/a | n/a |  |
| Post Primary Treatment | 5 | +12.68 (0.45 to 24.91), Ex | 74.2% |  |  | 5 | +10.03 (-2.65 to 22.71), Ex | 71.3% |  |
| Test For Subgroup Differences |  |  |  | n/a |  |  |  |  | n/a |
| **Follow-Up Duration** |  |  |  |  |  |  |  |  |  |
| 3-6 Months | - | - | - |  |  | 2 | +7.09 (-62.74 to 76.95), Ex | 37.2% |  |
| 7-12 Months | - | - | - |  |  | 3 | +10.67 (-21.89 to 43.40), Ex | 79.1% |  |
| >12 Months | - | - | - |  |  | - | - | - |  |
| Test For Subgroup Differences |  |  |  |  |  |  |  |  | 0.6962 |
| ^a^ Represents the MD in lower body strength (1repetition max, kg) between the exercise and control group at the end of the exercise intervention.  ^b^ Represents the MD in lower body strength (1repetition max, kg) between the exercise and control group at the end of the follow-up period.  Abbreviations: Ex, favors exercise | | | | | | | | | |

| **Table 5.** Treatment effect modifiers for changes in 6MWT distance. | | | | | | | | | |
| --- | --- | --- | --- | --- | --- | --- | --- | --- | --- |
| Intervention Modifier | **WMD** | | | | | | | | |
|  | **^a^Pre-Post** | | | |  | **^b^Pre-Follow Up** | | | |
|  | No. of Studies | Mean (95% CI), direction | I^2^ | p |  | No. of Studies | Mean (95% CI), direction | I^2^ | p |
| **Treatment Status** |  |  |  |  |  |  |  |  |  |
| No Modifier (Reference) | 8 | +40.31 (20.28 to 60.35), Ex | 41.2% |  |  | 8 | +49.41 (19.86 to 78.95), Ex | 68.6% |  |
| Active Treatment | 3 | +22.39 (-46.68 to 91.47), Ex | 66.9% |  |  | 3 | +25.74 (-83.92 to 135.40), Ex | 85.4% |  |
| Post Primary Treatment | 5 | +51.35 (38.97 to 63.74), Ex | 0.0% |  |  | 5 | +62.44 (38.94 to 85.93), Ex | 0.0% |  |
| Test For Subgroup Differences |  |  |  | 0.0822 |  |  |  |  | 0.1718 |
| **Follow-Up Duration** |  |  |  |  |  |  |  |  |  |
| 3-6 Months | - | - | - |  |  | 4 | +62.11 (38.12 to 86.10), Ex | 0.0% |  |
| 7-12 Months | - | - | - |  |  | 4 | +37.08 (-36.88 to 111.04), Ex | 80.7% |  |
| >12 Months | - | - | - |  |  | - | - | - |  |
| Test For Subgroup Differences |  |  |  |  |  |  |  |  | 0.3057 |
| ^a^ Represents the MD in VO_2_peak (ml/kg/min) between the exercise and control group at the end of the exercise intervention.  ^b^ Represents the MD in VO_2_peak (ml/kg/min) between the exercise and control group at the end of the follow-up period.  Abbreviations: Ex, favors exercise | | | | | | | | | |

| **Table 6.** Meta-regression results examining the association between follow-up duration (in months) and changes in physical performance. | | | | | | | | |
| --- | --- | --- | --- | --- | --- | --- | --- | --- |
|  | No. of Studies | Follow-Up Length (Months) | |  | Meta-Regression | | | |
|  |  | Mean ± SD | Range |  | β (95% CI) | R^2^ | I^2^ | p |
| VO_2peak_ (ml/kg/min) | 12 | 9.7 ± 14.8 | 3 to 56 |  | +0.02 (-0.12 to 0.14) | 0.00% | 78.7% | 0.77 |
| 6MWT (meters) | 8 | 6.3 ± 3.0 | 3 to 10 |  | -4.49 (-15.18 to 6.21) | 0.00% | 67.02% | 0.34 |
| 1RM Upper Body Strength(kg) | 5 | 8.2 ± 2.3 | 6 to 12 |  | -0.54 (-1.44 to 0.35) | 100% | 0.00% | 0.15 |
| 1RM Lower Body Strength (kg) | 5 | 8.0 ± 2.4 | 6 to 12 |  | -0.32 (-7.66 to 7.03) | 0.00% | 74.96% | 0.90 |
| β: Estimated change in outcome per 1-month increase in follow-up duration.  R^2^: Proportion of between-study variance explained by follow-up duration.  I^2^: Percentage of residual heterogeneity not explained by the model. | | | | | | | | |

**Figure 1.** Meta-regression of post-intervention and follow-up effects across outcomes. Panels show the association between post-intervention change and follow-up change for (A) VO₂peak, (B) upper-body strength, (C) lower-body strength, and (D) 6MWT performance. Lines represent the estimated meta-regression slope with 95% confidence intervals.

Figure 1. Funnel plot results for Vo2peak

Assessment of publication bias (e.g., funnel plots, Egger’s test) was conducted only for cardiorespiratory fitness, as all other analyses included fewer than 10 studies. With small study numbers, such tests are not recommended due to low statistical power and the potential for misleading results [7].

A) Funnel plot for pre to post intervention effects for Vo2peak. Egger’s test indicated no evidence of small-study effects (p = 0.92).

**A)**

B) Funnel plot for follow-up effects for Vo2peak. Egger’s test indicated no evidence of small-study effects (p = 0.52).

**B)**

**Table 7. GRADE Certainty of Evidence Profile**

**Question:** Exercise compared to no exercise for physical function in cancer survivors

| **Certainty assessment** | | | | | | | **№ of patients** | | **Effect** | | **Certainty** | **Importance** |
| --- | --- | --- | --- | --- | --- | --- | --- | --- | --- | --- | --- | --- |
| **№ of studies** | **Study design** | **Risk of bias** | **Inconsistency** | **Indirectness** | **Imprecision** | **Other considerations** | **exercise** | **no exercise** | **Relative (95% CI)** | **Absolute (95% CI)** |  |  |
| **VO2peak (follow-up: mean 9.7 months)** | | | | | | | | | | | | |
| 12 | randomised trials | serious^a^ | serious^b^ | not serious | very serious^c^ | none | 353 | 276 | - | MD **1.24 ml/kg/min higher** (0.43 lower to 2.9 higher) | ⨁◯◯◯ Very low^a,b,c^ | IMPORTANT |
| **Upper Body Strength (1RM) (follow-up: mean 8.2 months)** | | | | | | | | | | | | |
| 5 | randomised trials | serious^a^ | not serious | not serious | very serious^d^ | none | 161 | 147 | - | MD **1.8 kg higher** (0.36 lower to 3.95 higher) | ⨁◯◯◯ Very low^a,d^ | IMPORTANT |
| **Lower Body Strength (1RM) (follow-up: mean 8 months)** | | | | | | | | | | | | |
| 5 | randomised trials | serious^a^ | serious^b^ | not serious | very serious^e^ | none | 233 | 215 | - | MD **10.3 kg higher** (2.65 lower to 22.71 higher) | ⨁◯◯◯ Very low^a,b,e^ | IMPORTANT |
| **6 Minute Walk Test (6MWT) (follow-up: mean 6.3 months)** | | | | | | | | | | | | |
| 8 | randomised trials | serious^a^ | serious^b^ | not serious | not serious | none | 326 | 315 | - | MD **49.41 meters higher** (19.86 higher to 78.95 higher) | ⨁⨁◯◯ Low^a,b^ | IMPORTANT |

**CI:** confidence interval; **MD:** mean difference

#### Explanations

a. Rated as serious risk of bias due to substantial loss to follow-up (30% on average).

b. Significant heterogeneity exists between studies (I2 > 50%).

c. The 95% confidence interval crosses the TE threshold of +1.35ml/kg/min and 0 ml/kg/min.

d. The 95% confidence interval crossed the threshold of +1.8kg and 0kg.

e. The 95% confidence interval crossed the threshold of +5.5kg and 0kg.

| **Table 8.** Sensitivity analysis of post WMD estimates using SDs of change scores calculated with correlation coefficients (r) of 0.5, 0.7, and 0.9. The primary analysis used r = 0.7. | | | | | | | | | | | |
| --- | --- | --- | --- | --- | --- | --- | --- | --- | --- | --- | --- |
|  | r = 0.5 | | |  | r = 0.7 | | |  | r = 0.9 | | |
|  | Mean (95% CI) | p | I^2^ |  | Mean (95% CI) | p | I^2^ |  | Mean (95% CI) | p | I^2^ |
| VO_2peak_ (ml/kg/min) | +1.75 (0.51 to 2.99) | 0.0100 | 60.4% |  | +1.76 (0.55 to 2.96) | 0.0083 | 66.8% |  | +1.80 (0.64 to 2.95) | 0.0057 | 79.2% |
| 6MWT (meters) | +39.04 (19.36 to 58.73) | 0.0022 | 25.8% |  | +40.31 (20.28 to 60.35) | 0.0021 | 41.2% |  | +42.90 (21.39 to 64.42) | 0.0022 | 69.3% |
| 1RM Upper Body Strength (kg) | +3.27 (2.21 to 4.32) | 0.001 | 0.0% |  | +3.35 (2.16 to 4.53) | 0.0014 | 0.0% |  | +3.50 (2.14 to 4.86) | 0.0020 | 3.8% |
| 1RM Lower Body Strength (kg) | +12.50 (0.52 to 24.49) | 0.0443 | 60.5% |  | +12.68 (0.45 to 24.91) | 0.0450 | 74.2% |  | +12.52 (0.16 to 24.89) | 0.0482 | 88.6% |

| **Table 9.** Sensitivity analysis of follow-up WMD estimates using SDs of change scores calculated with correlation coefficients (r) of 0.5, 0.7, and 0.9. The primary analysis used r = 0.7. | | | | | | | | | | | |
| --- | --- | --- | --- | --- | --- | --- | --- | --- | --- | --- | --- |
|  | r = 0.5 | | |  | r = 0.7 | | |  | r = 0.9 | | |
|  | Mean (95% CI) | p | I^2^ |  | Mean (95% CI) | p | I^2^ |  | Mean (95% CI) | p | I^2^ |
| VO_2peak_ (ml/kg/min) | +1.19 (-0.53 to 2.92) | 0.1554 | 73.7% |  | +1.24 (-0.43 to 2.90) | 0.1300 | 76.1% |  | +1.31 (-0.25 to 2.88) | 0.0909 | 81.8% |
| 6MWT (meters) | +47.81 (18.26 to 77.36) | 0.0065 | 58.8% |  | +49.41 (19.86 to 78.95) | 0.0055 | 68.6% |  | +51.21 (21.83 to 80.59) | 0.0045 | 83.7% |
| 1RM Upper Body Strength (kg) | +1.84 (-0.11 to 3.78) | 0.0589 | 0.0% |  | +1.80 (-0.36 to 3.95) | 0.0814 | 4.7% |  | +1.84 (-0.60 to 4.29) | 0.1037 | 66.4% |
| 1RM Lower Body Strength (kg) | +9.80 (-2.54 to 22.15) | 0.0922 | 56.7% |  | +10.03 (-2.65 to 22.71) | 0.0930 | 71.3% |  | + 10.01 (-2.97 to 22.99) | 0.0990 | 87.8% |

**References**

1.	Anandavadivelan P, Mijwel S, Wiklander M, Kjoe PLM, Luijendijk M, Bergh J, Rundqvist H, Wengstrom Y: Five-year follow-up of the OptiTrain trial on concurrent resistance and high-intensity interval training during chemotherapy for patients with breast cancer. *Scientific Reports* 2024, 14(1):15333.

2.	Van Vulpen JK, Velthuis MJ, Steins Bisschop CN, Travier N, Van Den Buijs BJ, Backx FJ, Los M, Erdkamp FL, Bloemendal HJ, Koopman M *et al*: Effects of an Exercise Program in Colon Cancer Patients undergoing Chemotherapy. *Med Sci Sports Exerc* 2016, 48(5):767-775.

3.	Rasmussen GHF, Kristiansen M, Arroyo-Morales M, Voigt M, Madeleine P: The Analgesic Effect of Resistance Training after Breast Cancer (ANTRAC): A Randomized Controlled Trial. *Medicine & Science in Sports & Exercise* 2023, 55(2):167-176.

4.	Winters-Stone KM, Horak F, Dieckmann NF, Luoh SW, Eckstrom E, Stoyles SA, Roeland EJ, Li F: GET FIT: A Randomized Clinical Trial of Tai Ji Quan Versus Strength Training for Fall Prevention After Chemotherapy in Older, Postmenopausal Women Cancer Survivors. *J Clin Oncol* 2023, 41(18):3384-3396.

5.	Rogers LQ, Courneya KS, Oster RA, Anton PM, Phillips S, Ehlers DK, McAuley E: Physical activity intervention benefits persist months post-intervention: randomized trial in breast cancer survivors. *J Cancer Surviv* 2023, 17(6):1834-1846.

6.	Vehmanen L, Sievänen H, Kellokumpu-Lehtinen P, Nikander R, Huovinen R, Ruohola J, Penttinen HM, Utriainen M, Tokola K, Blomqvist C *et al*: Five-year follow-up results of aerobic and impact training on bone mineral density in early breast cancer patients. *Osteoporos Int* 2021, 32(3):473-482.

7.	Chandler J, Cumpston M, Li T, Page MJ, Welch V: Cochrane handbook for systematic reviews of interventions. *Hoboken: Wiley* 2019, 4(1002):14651858.
